# Prevalence of Polypharmacy, Hyperpolypharmacy and Potentially Inappropriate Medication Use in Older Adults in India: A Systematic Review and Meta-Analysis

**DOI:** 10.3389/fphar.2021.685518

**Published:** 2021-05-19

**Authors:** Akshaya S. Bhagavathula, Kota Vidyasagar, Manik Chhabra, Muhammed Rashid, Rishabh Sharma, Deepak K. Bandari, Daniela Fialova

**Affiliations:** ^1^ Department of Social and Clinical Pharmacy, Faculty of Pharmacy in Hradec Kralove, Charles University, Hradec Kralove, Czechia; ^2^ University College of Pharmaceutical Sciences, Kakatiya University, Warangal, India; ^3^ Indo-Soviet Friendship College of Pharmacy, Moga, India; ^4^ Department of Pharmacy Practice, Sri Adichunchanagiri College of Pharmacy, Mandya, India; ^5^ Department of Geriatrics and Gerontology, 1st Faculty of Medicine, Charles University, Prague, Czechia

**Keywords:** polypharmacy (source: MeSH, NML), India, potentially inappropriate medication (PIM), prevalence, older (diseased) population, hyperpolypharmacy

## Abstract

**Background:** Older people often receive multiple medications for chronic conditions, which often result in polypharmacy (concomitant use of 5‒9 medicines) and hyperpolypharmacy (concomitant use of ≥10 medicines). A limited number of studies have been performed to evaluate the prevalence of polypharmacy, hyperpolypharmacy, and potentially inappropriate medication (PIM) use in older people of developing countries. The present study aimed to investigate regional variations in the prevalence of polypharmacy, hyperpolypharmacy, and PIM use in older people (60 + years) in India.

**Methods:** Studies were identified using Medline/PubMed, Scopus, and Google Scholar databases published from inception (2002) to September 31, 2020. Out of the total 1890 articles, 27 were included in the study.

**Results:** Overall, the pooled prevalence of polypharmacy was 49% (95% confidence interval: 42–56; *p* < 0.01), hyperpolypharmacy was 31% (21–40; *p* < 0.01), and PIM use was 28% (24–32; *p* < 0.01) among older Indian adults. Polypharmacy was more prevalent in North-east India (65%, 50–79), whereas hyperpolypharmacy was prevalent in south India (33%, 17–48). Region-wize estimates for the pooled prevalence of PIM use in India were as follows: 23% (21–25) in East, 33% in West (24–42), 17.8% in North (11–23), and 32% (26–38) in South India. The prevalence of PIM use in adults aged ≥70°years was 35% (28–42), in those taking more medications (≥5.5/day) was 27% (22–31), and in adults using a high number of PIMs (≥3) was 29% (22–36). Subgroup analysis showed that cross-sectional studies had a higher pooled prevalence of polypharmacy 55% (44–65) than cohorts 45% (37–54). Hyperpolypharmacy in inpatient care settings was 37% (26–47), whereas PIM use was higher in private hospitals 31% (24–38) than government hospitals 25% (19–31).

**Conclusion:** Polypharmacy and hyperpolypharmacy are widely prevalent in India. About 28% of older Indian adults are affected by PIM use. Thus, appropriate steps are needed to promote rational geriatric prescribing in India.

**Systematic Review Registration**: https://clinicaltrials.gov, identifier [CRD42019141037].

## Introduction

There were 703 million people aged 65°years or over in the world in 2019. The number of the older people is projected to double to 1.5 billion by 2050, with a more prominent increase in developing countries (He and Kinsella, 2020). According to the United Nations Population Fund’s (UNFPA) 2021 flagship State of World population report, there are nearly 93 million (6.8%) older people (aged 65°years or above) in India, and the number is projected to exceed 227 million in 2050 (United Nations Population Fund).

In general, older people often receive multiple medications for chronic conditions, which often result in polypharmacy (concomitant use of 5‒9 medicines) and hyperpolypharmacy (concomitant use of ≥10 medicines) (Masnoon et al., 2017). Research shows that older adults in India frequently use multiple medications. There are wide regional variations in the prevalence ranging from 5.8% in West Bengal (west region) and 93.1% in Uttaranchal (North India) (Sharma et al., 2019). Although medications are essential to improve a patient’s health status and quality of life, suboptimal prescribing and the use of multiple drugs may have adverse outcomes (Pravodelov, 2020; O’Mahony, 2020; Bala et al., 2019). Moreover, polypharmacy and hyperpolypharmacy are strongly linked to a broad range of negative health outcomes and are considered proxy indicators of potentially inappropriate medication (PIM) use (Guillot et al., 2020).

The term PIM is defined as medications that have adverse effects and, when used by older adults, may outweigh the clinical advantages of the drug, such as mental and functional decline, adverse drug events, drug interactions, unplanned hospitalization, morbidity, and mortality (Thomas and Thomas, 2019; Xing et al., 2019; de Oliveira et al., 2020; Weeda et al., 2020). Higher-income countries have taken several steps to improve rational prescribing in older adults and have developed evidence-based explicit tools to screen and prevent PIM use in older patients. Explicit tools comprise lists of drugs or drug classes (developed from literature reviews, expert opinion, and consensus techniques) that, when prescribed or underprescribed, can cause harm in older people. Beers criteria and the Screening Tool of Older Persons’ prescription (STOPP) and the Screening Tool to Alert to Right Treatment (START) are the most commonly referenced tools (Topinková et al., 2008; Hill-Taylor et al., 2013; American Geriatrics Society 2015 Beers Criteria Update Expert Panel, 2015; Thomas and Thomas, 2019; Weeda et al., 2020).

Several systematic reviews and meta-analyses on the prevalence of polypharmacy and PIM use in the older population, using data from developed countries (Kaufmann et al., 2014; Muhlack et al., 2017; Liew et al., 2019; Thomas and Thomas, 2019; Xing et al., 2019; Davies et al., 2020; de Oliveira et al., 2020; Liew et al., 2020; Mohamed et al., 2020; Weeda et al., 2020), indicated a rising trend of inappropriate medication use in the current healthcare system. However, differences in the population, healthcare settings, and medication use process may limit the generalizability of these findings in developing countries, including India. Given the rapidly increasing older population, increasing burden of chronic diseases, and wide variations in polypharmacy use across India, the prevalence of PIM use in the Indian older population is pertinent. We hypothesized that the prevalence of polypharmacy, hyperpolypharmacy, and PIM use in India would be higher than in the western countries, and their distribution may vary across different states in India. Thus, this study aimed to perform a systematic review and meta-analysis to assess the overall prevalence and regional variations (north, east, west, and south: NEWS) of polypharmacy, hyperpolypharmacy, and PIM use in older people in India.

## Methods

The study was performed according to the MOOSE (Meta-analysis of Observational Studies in Epidemiology) guidelines (Stroup et al., 2000). The research protocol is registered on PROSPERO, 2019 (CRD42019141037).

### Search Strategy

We comprehensively searched Medline/PubMed, Scopus, Google Scholar, and bibliographic databases from inception (2002) to September 31, 2020. The search process was initiated in april 2019 and updated until September 31, 2020. We used combinations of Medical Subject Headings (MeSH) and free text words to identify the relevant studies related to the exposure (e.g., polypharmacy, hyperpolypharmacy, potentially inappropriate prescribing (PIP), PIMs, and to search terms related to outcomes (e.g., prevalence, estimates, percentage, burden). Complete details about the search terms used in various databases have been listed in Supplementary Table S1.

### Selection Criteria and Data Extraction

The studies met the following criteria; observational (cross-sectional, case-cohort, or cohort) on the older population (aged 60 and older), conducted in India, and reported prevalence of polypharmacy, hyperpolypharmacy, and PIM use, using any explicit criteria to assess the appropriateness of drugs prescribed. The following articles were excluded; duplicate studies, abstracts, letters, editorials, conference proceedings, review articles, meta-analyses, non-population-based studies, and interventional studies.

### Selection of Studies

Three reviewers (ASB, RS and KVS) independently screened the titles and abstracts of the initially identified studies to determine whether each study met the predefined eligibility criteria. Full-text articles were retrieved for selected titles. References of the retrieved articles were also screened to identify the additional eligible articles. Any disagreements regarding selection were resolved through discussion, consensus, or consultation with other team authors (MC, MR, and SPS).

### Data Extraction

Full texts of included studies were read, and three reviewers (RS, MC, and KVS) extracted the relevant data from the selected studies. The extracted data included author details, year of publication, geographic origin, study design and settings, patient sampling, participant characteristics (e.g., age range, mean age, sex, comorbidities, and number of prescribed medications), measurements (explicit criteria), and information on outcomes (type of medication use, number of patients exposed to PIM, number of PIMs identified and percentage of the older population on polypharmacy and hyperpolypharmacy). Prevalence estimates of PIM use were stratified to provide specific estimates of the subsets (mean age, gender, study duration, and the average number of medications).

### Quality Assessment

The methodological quality of the included studies was evaluated using the Newcastle Ottawa Scale (NOS) for cross-sectional and cohort studies (Luchini et al., 2017). The NOS assesses the representativeness of the sample, sample size, response rate, ascertainment of exposure, control of confounding variables, assessment of preventability, and appropriate statistical analysis. The NOS scores range from 0 (lowest grade) to 9 (highest grade). Studies scoring seven or above were considered high quality, and those with scores below seven were of low quality.

### Statistical Analysis

The estimates of polypharmacy, hyperpolypharmacy, and PIM use were expressed as proportions (%) with corresponding 95% confidence intervals (CI). The pooled prevalence estimates of outcome variables were calculated using regional population size weights. The magnitude of heterogeneity between the studies was assessed using the *I* ^*2*^ statistic (% residual variation due to heterogeneity), and Tau^2^ (method of moments estimate of between-study variance) was used for each of the pooled estimates. *I* ^*2*^ values range between 0 and 100%, and is considered low for *I* ^*2*^ <25%, modest for 25–50%, and large for >50% (Higgins et al., 2003). As differences between the studies were very high (95–99% inconsistency), a random effect DerSimonian-Laird model was used in all analyses (Higgins et al., 2003). In case of substantial heterogeneity, the source of heterogeneity was investigated using stratified analyses and meta-regression analysis, based on the study-level characteristics, such as year of publication, study duration, mean age, women-to-men ratio, the mean number of drugs, number of PIM use, and quality of studies based on NOS scale. The interaction between the subgroups of each factor was assessed using Cochran's *Q* test, degree of freedom (df), and *p*-value resulting from Cochran’s *Q* test. A *p*-value of <0.10 was considered statistically significant for Cochran’s *Q* test (Huedo-Medina et al., 2006). The risk of publication bias was inspected by using the symmetry of funnel plots, and Egger’s and Begg’s tests were also used. Statistical analyses were performed using STATA software, version 16 MP (StataCorp, College Station, TX).

## Results

### Study Selection

**FIGURE 1:**
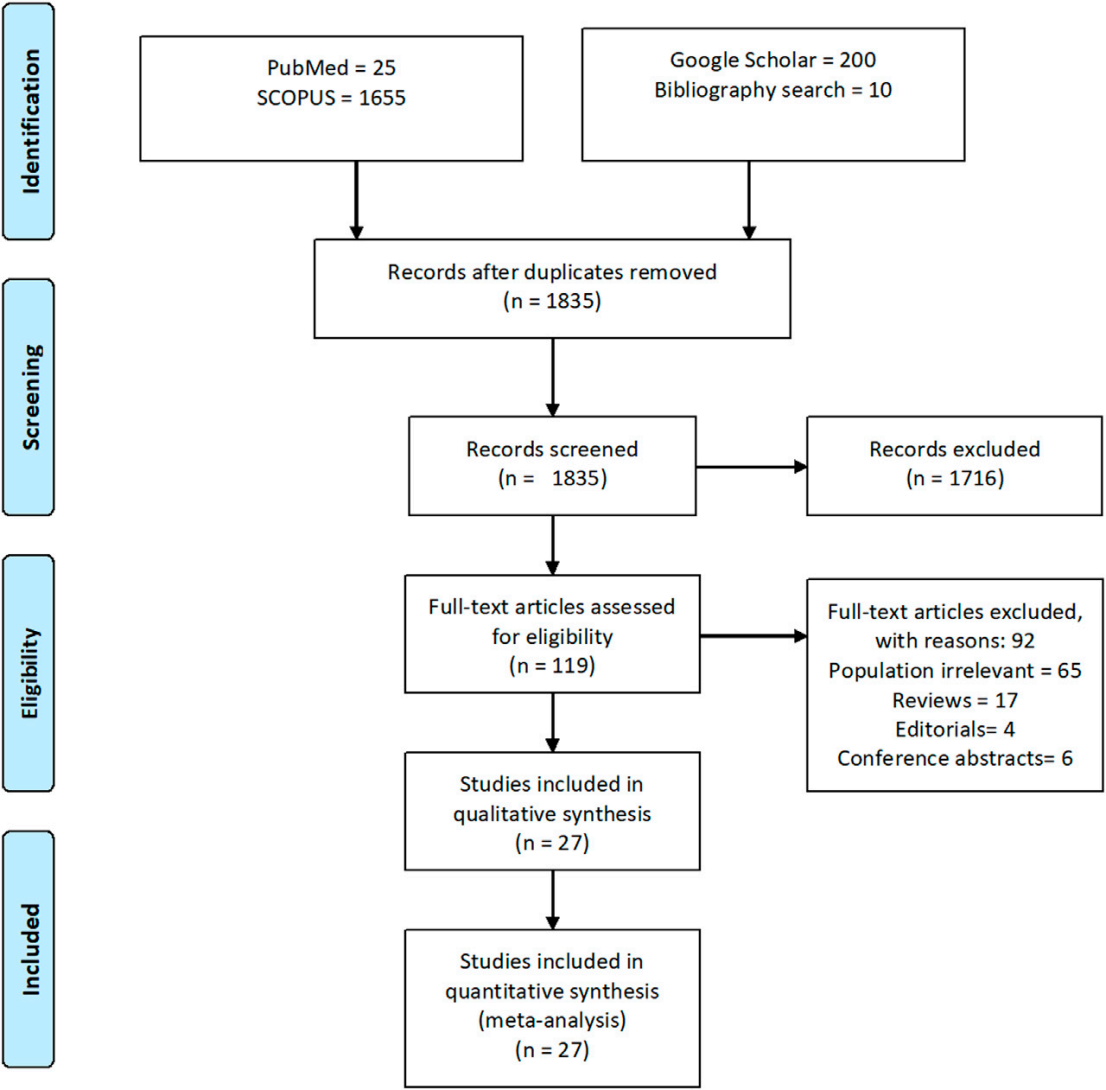
PRISMA diagram of the literature selection in this systematic literature review and meta-analysis.

A total of 1890 references were initially identified through electronic databases. After removing 165 duplicates, a total of 1835 titles and abstracts were screened to determine if they met the inclusion criteria, as described in the methodology section. Full-text assessment of 119 potentially relevant articles resulted in 27 eligible studies (Bhatt et al., 2019; Chandrasekhar et al., 2019; Motallebzadeh et al., 2019; Pradhan and Panda, 2018; Benjamin et al., 2018; Devarapalli et al., 2017; Kumar et al., 2017; Pradhan et al., 2017; Narvekar et al., 2017; Borah et al., 2017; Rakesh et al., 2017; Anjum et al., 2017; Swathi and Bhavika, 2016; Salwe et al., 2016; Chowta et al., 2016; Kashyap et al., 2015; Danisha et al., 2015; Umar et al., 2015; Undela et al., 2014; Dhikav et al., 2014; Karandikar et al., 2013; Momin et al., 2013; Nagendra et al., 2012; Shah et al., 2011; Mandavi et al., 2011; Zaveri et al., 2010; Harugeri et al., 2010), as shown in Figure 1. The list of articles that are excluded (*n* = 92) due to various reasons is presented in Supplementary Table S2.

### Characteristics of Included Studies

All the studies included in the present study were published between 2010 and 2019. Sample size varied on regional basis from 90 to 1,510, making a total of 11,649 patients. All the studies included both women and men (Bhatt et al., 2019; Chandrasekhar et al., 2019; Motallebzadeh et al., 2019; Pradhan and Panda, 2018; Benjamin et al., 2018; Devarapalli et al., 2017; Kumar et al., 2017; Pradhan et al., 2017; Narvekar et al., 2017; Borah et al., 2017; Rakesh et al., 2017; Anjum et al., 2017; Swathi and Bhavika, 2016; Salwe et al., 2016; Chowta et al., 2016; Kashyap et al., 2015; Danisha et al., 2015; Umar et al., 2015; Undela et al., 2014; Dhikav et al., 2014; Karandikar et al., 2013; Momin et al., 2013; Nagendra et al., 2012; Shah et al., 2011; Mandavi et al., 2011; Zaveri et al., 2010; Harugeri et al., 2010); however, seven studies included more women than men(Dhikav et al., 2014; Umar et al., 2015; Salwe et al., 2016; Kumar et al., 2017; Narvekar et al., 2017; Bhatt et al., 2019; Chandrasekhar et al., 2019). Among the studies, nineteen were cohort design (Chandrasekhar et al., 2019; Motallebzadeh et al., 2019; Pradhan and Panda, 2018; Benjamin et al., 2018; Devarapalli et al., 2017; Pradhan et al., 2017; Narvekar et al., 2017; Anjum et al., 2017; Salwe et al., 2016; Chowta et al., 2016; Kashyap et al., 2015; Danisha et al., 2015; Umar et al., 2015; Undela et al., 2014; Dhikav et al., 2014; Karandikar et al., 2013; Nagendra et al., 2012; Shah et al., 2011; Harugeri et al., 2010), and eight were cross-sectional studies (Zaveri et al., 2010; Mandavi et al., 2011; Momin et al., 2013; Swathi and Bhavika, 2016; Borah et al., 2017; Kumar et al., 2017; Rakesh et al., 2017; Bhatt et al., 2019). The majority of the studies were conducted in South India (Bhatt et al., 2019; Chandrasekhar et al., 2019; Motallebzadeh et al., 2019; Benjamin et al., 2018; Rakesh et al., 2017; Anjum et al., 2017; Swathi and Bhavika, 2016; Salwe et al., 2016; Chowta et al., 2016; Danisha et al., 2015; Umar et al., 2015; Nagendra et al., 2012; Harugeri et al., 2010), six in North India (Mandavi et al., 2011; Karandikar et al., 2013; Dhikav et al., 2014; Undela et al., 2014; Kashyap et al., 2015; Kumar et al., 2017), three in Eastern states (Devarapalli et al., 2017; Pradhan et al., 2017; Pradhan and Panda, 2018), four in Western region (Zaveri et al., 2010; Shah et al., 2011; Momin et al., 2013; Narvekar et al., 2017) and only one study in North-east India (Borah et al., 2017). Twenty-one studies provided data on the prevalence of polypharmacy (Bhatt et al., 2019; Chandrasekhar et al., 2019; Motallebzadeh et al., 2019; Pradhan and Panda, 2018; Benjamin et al., 2018; Devarapalli et al., 2017; Pradhan et al., 2017; Borah et al., 2017; Rakesh et al., 2017; Anjum et al., 2017; Swathi & Bhavika, 2016; Salwe et al., 2016; Chowta et al., 2016; Kashyap et al., 2015; Umar et al., 2015; Undela et al., 2014; Karandikar et al., 2013; Momin et al., 2013; Nagendra et al., 2012; Mandavi et al., 2011; Harugeri et al., 2010), fourteen studies reported estimates of hyperpolypharmacy (Bhatt et al., 2019; Chandrasekhar et al., 2019; Benjamin et al., 2018; Devarapalli et al., 2017; Anjum et al., 2017; Salwe et al., 2016; Chowta et al., 2016; Kashyap et al., 2015; Umar et al., 2015; Undela et al., 2014; Karandikar et al., 2013; Momin et al., 2013; Nagendra et al., 2012; Harugeri et al., 2010), whereas all the twenty-seven studies reported PIM use in the older population (Bhatt et al., 2019; Chandrasekhar et al., 2019; Motallebzadeh et al., 2019; Pradhan and Panda, 2018; Benjamin et al., 2018; Devarapalli et al., 2017; Kumar et al., 2017; Pradhan et al., 2017; Narvekar et al., 2017; Borah et al., 2017; Rakesh et al., 2017; Anjum et al., 2017; Swathi and Bhavika, 2016; Salwe et al., 2016; Chowta et al., 2016; Kashyap et al., 2015; Danisha et al., 2015; Umar et al., 2015; Undela et al., 2014; Dhikav et al., 2014; Karandikar et al., 2013; Momin et al., 2013; Nagendra et al., 2012; Shah et al., 2011; Mandavi et al., 2011; Zaveri et al., 2010; Harugeri et al., 2010). Most of the studies used 2012 Beers criteria (Bhatt et al., 2019; Motallebzadeh et al., 2019; Pradhan and Panda, 2018; Devarapalli et al., 2017; Kumar et al., 2017; Pradhan et al., 2017; Narvekar et al., 2017; Borah et al., 2017; Anjum et al., 2017; Swathi and Bhavika, 2016; Salwe et al., 2016; Kashyap et al., 2015; Danisha et al., 2015; Umar et al., 2015; Dhikav et al., 2014), only one study used 2015 STOPP/START criteria (Chandrasekhar et al., 2019), while the rest of the studies used a different version of the Beers criteria in combination with other PIM criteria (Shah et al., 2011; Nagendra et al., 2012; Karandikar et al., 2013; Chowta et al., 2016; Rakesh et al., 2017; Benjamin et al., 2018). The characteristics of the included studies are summarized in Table 1.

**TABLE 1:** Characteristics of included studies.

Author, year	Study characteristics						Explicit criteria	Prevalence (%)		
	States	Design	Period	Setting	Sample size	Age, years (Mean/median)	Explicit criteria	Polypharmacy^a^	Hyperpolypharmacy^b^	PIM use
Bhatt et al. (2019)	Kerala	Cross-sectional	6	Outpatient	400	73.6 ± 6.7	Beer's criteria	45.8	13.5	34
Chandrasekhar et al. (2019)	Kerala	Cohort	12	Inpatient	210	Phase 1: 72.59 ± 6.37	STOPP/START criteria	60	35.7	Overall: 41.9, phase 1: 43.5, phase 2: 40.2
						Phase 2: (71.99 ± 6.30				
Motallebzadeh et al., (2019)	Karnataka	Cohort	6	Inpatient	480	Unspecified	Beers criteria	36.4	Unspecified	11.6
Benjamin et al. (2018)	Karnataka	Cohort	7	Inpatient	350	92 (68)	Beers criteria, STOPP criteria	37.1	58.6	2012 Beers: 27.7, STOPP: 24.6
Pradhan et al. (2018)	Odisha	Cross-sectional	3	Outpatient	425	72.5 ± 7.6	Beers criteria	75.1	Unspecified	23.8
Devarapalli et al. (2017)	Andhra Pradesh	Cohort	Unspecified	Inpatient	135	66.9 ± 0.2	Beers criteria	38.5	35.5	25.9
Kumar et al., (2017)	Jammu & kashmir	Cohort	6	Inpatient	203	Unspecified	Beers criteria	Unspecified	Unspecified	3.7
Pradhan et al. (2017)	Odisha	Cross-sectional	4	Outpatient	800	75.8 ± 6.9	Beers criteria	41.5	Unspecified	21.8
Narvekar et al. (2017)	Goa	Cohort	5	Inpatient	150	68.88 (range: 60–87)	Beers criteria	Unspecified	Unspecified	44
Borah et al. (2017)	Assam	Cross-sectional	6	Both	150	Unspecified	Beers criteria	72	Unspecified	28.7
Rakesh et al. (2017)	Karnataka	Cross-sectional	16	Outpatient	426	71.6 ± 6.4	MAI, beers criteria, STOPP criteria, and START criteria	66.2	Unspecified	19.9
Anjum et al. (2017)	Tamil nadu	Cohort	6	Inpatient	90	Unspecified	Beers criteria	40	50	51.1
Burla et al. (2016)	Telangana	Cohort	3	Outpatient	287	Unspecified	Beers criteria	68.3	Unspecified	20.2
Salwe et al. (2016)	Puducherry	Cross-sectional	3	Inpatient	100	71.64 ± 6.51	Beers criteria	53	27	48
Chowta et al. (2016)	Karnataka	Cross-sectional	12	Outpatient	120	71.56 ± 6.61	Medication appropriateness index, STOPP/START, Beer’s criteria	42.5	2.5	32.5
Kashyapa et al. (2015)	Chandigarh	Cohort	Unspecified	Inpatient	1,510	67.2 ± 0.2	Beers criteria	39	38.7	21
Pattani et al. (2015)	Kerala	Cohort	12	Inpatient	200	72.2 ± 8.04	Beers criteria	Unspecified	Unspecified	53
Umar et al. (2015)	Karnataka	Cohort	6	Inpatient	203	70 ± 2.4	Beers criteria	57.1	7.9	37.4
Undela et al. (2013)	Chandigarh	Cohort	9	Inpatient	1,215	68 ± 7.0	Beers criteria 2003 and beers criteria 2012	46	40	2003 Beers: 11
										2012 Beers: 16
Dhikav et al. (2014)	New Delhi	Cohort	12	Outpatient	143	70.1 ± 10.1	Beers criteria	Unspecified	Unspecified	41.9
Karandikar et al. (2013)	Maharashtra	Cross-sectional	8	Both	600	Unspecified	Beers criteria and STOPP/START criteria	41	15	STOPP: 11.9 Beers: 7.3
Momin et al. (2013)	Gujarat	Cohort	12	Inpatient	210	69.34 ± 5.26	Beers criteria 2003 and 2012	50.9	34.7	2003 Beers: 40 2012 Beers: 28.57
Vishwas et al. (2012)	Karnataka	Cohort	9	Inpatient	540	66 (range: 60–95)	Beers criteria and STOPP	50.2	44.4	24.6
Shah et al. (2011)	Gujarat	Cohort	27	Both	400	Unspecified	Beers criteria and Phadke’s criteria	Unspecified	Unspecified	27.2
Mandavi et al. (2011)	Chandigarh	Cohort	5	Outpatient	1,081	68.2 ± 0.20	Beers criteria	58	Unspecified	10.8
Zaveri et al. (2010)	Gujarat	Cohort	4	Outpatient	407	Unspecified	Beers criteria	Unspecified	Unspecified	23.6
Harugeri et al. (2010)	Karnataka	Cohort	18	Inpatient	814	66 years (range: 60–95)	Beers criteria	36.6	53.7	23.5

a using >5 drugs;

b using ≥10 drugs, PIM: potentially inappropriate medication; STOPP: screening tool of older persons’ prescriptions; START: screening tool to alert to right treatment.

### Quality of Included Studies

The quality assessment of included studies was assessed using NOS for the cross-sectional and cohort studies. The highest quality score was 9, and the lowest was 4. The average score of the NOS scale was 7.4, indicating high quality. In the risk of bias assessment, four studies (14.8%) were of lower quality, with a NOS score of <7. Based on NOS criteria, three studies were of lower quality based on criteria 1 (representativeness of the exposure group or sample representation), seven studies based on criteria 2 (selection of non-exposure group or sample selection), and three studies based on criteria 3 (not report the definition of the exposure); only four of eight cross-sectional studies performed appropriate statistical tests (criteria 7). Detailed results on the NOS quality assessment are presented in Supplementary Table S3.

### Prevalence of Polypharmacy

**FIGURE 2:**
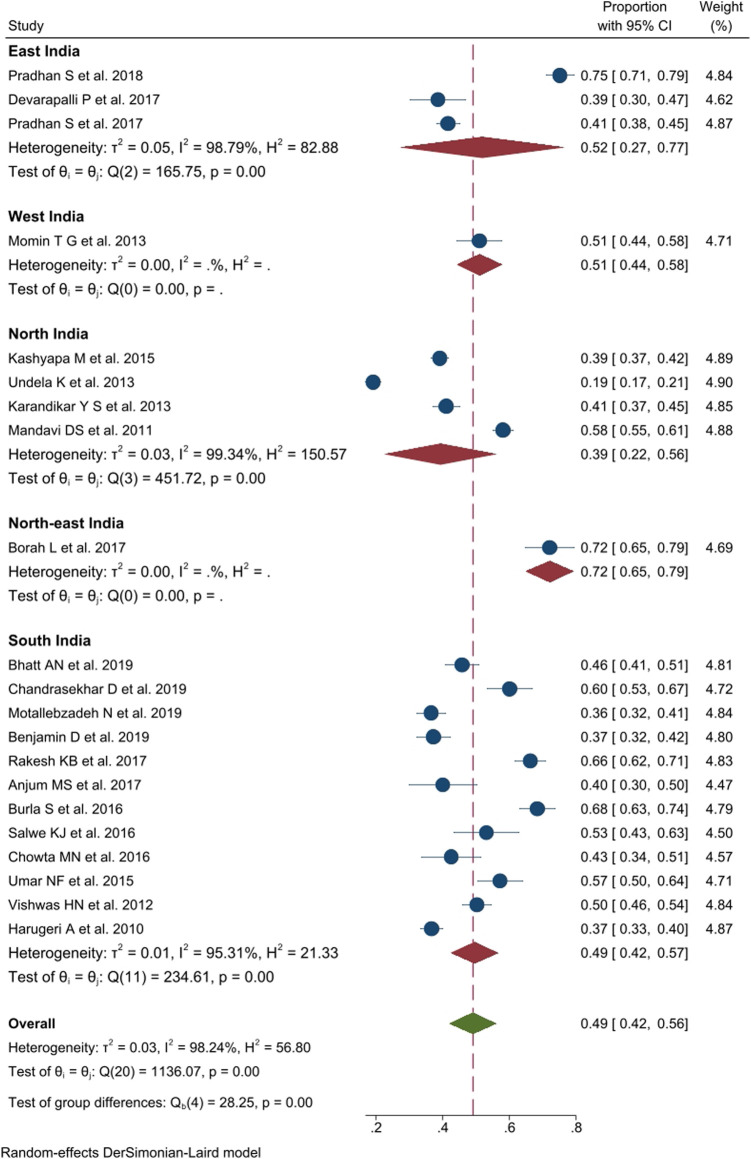
Prevalence of polypharmacy use (5-9 medications) in older people across various geographic regions in India.

Out of 27 publications, twenty-one studies, comprising 9,391 participants, reported a prevalence of polypharmacy among older adults. The pooled prevalence of polypharmacy in India, after weighing the regional population size, was 49% (*n* = 10,146, 95% CI: 42–56; *I* ^*2*^ = 98.2%, *p* < 0.01, τ^2^ 0.03). Region-wize data showed significant differences in the prevalence of polypharmacy between different regions of India (*Q* = 5.47, df = 4; *p* < 0.01) ranging from 39% (95% CI: 22–56; *I* ^*2*^ = 99.3%, *p* < 0.01) in Northern states to 52% (95% CI: 27–77, *I* ^*2*^ = 98.8%, *p* < 0.01) in East India. Studies from West India (51%, 95% CI: 44–58), and North-east India reported higher prevalence of polypharmacy (72%, 95% CI: 65–79). Moreover, the majority of studies were conducted in South India (Harugeri et al., 2010; Nagendra et al., 2012; Kashyap et al., 2015; Umar et al., 2015; Chowta et al., 2016; Salwe et al., 2016; Swathi and Bhavika, 2016; Devarapalli et al., 2017; Narvekar et al., 2017; Pradhan and Panda, 2018; Bhatt et al., 2019; Chandrasekhar et al., 2019; Motallebzadeh et al., 2019), where the prevalence of polypharmacy was 49% (95% CI: 42–57; *I* ^*2*^ = 95.3%, *p* < 0.01). The data on the prevalence of polypharmacy in other regions is summarized in Figure 2.

### Hyperpolypharmacy

**FIGURE 3:**
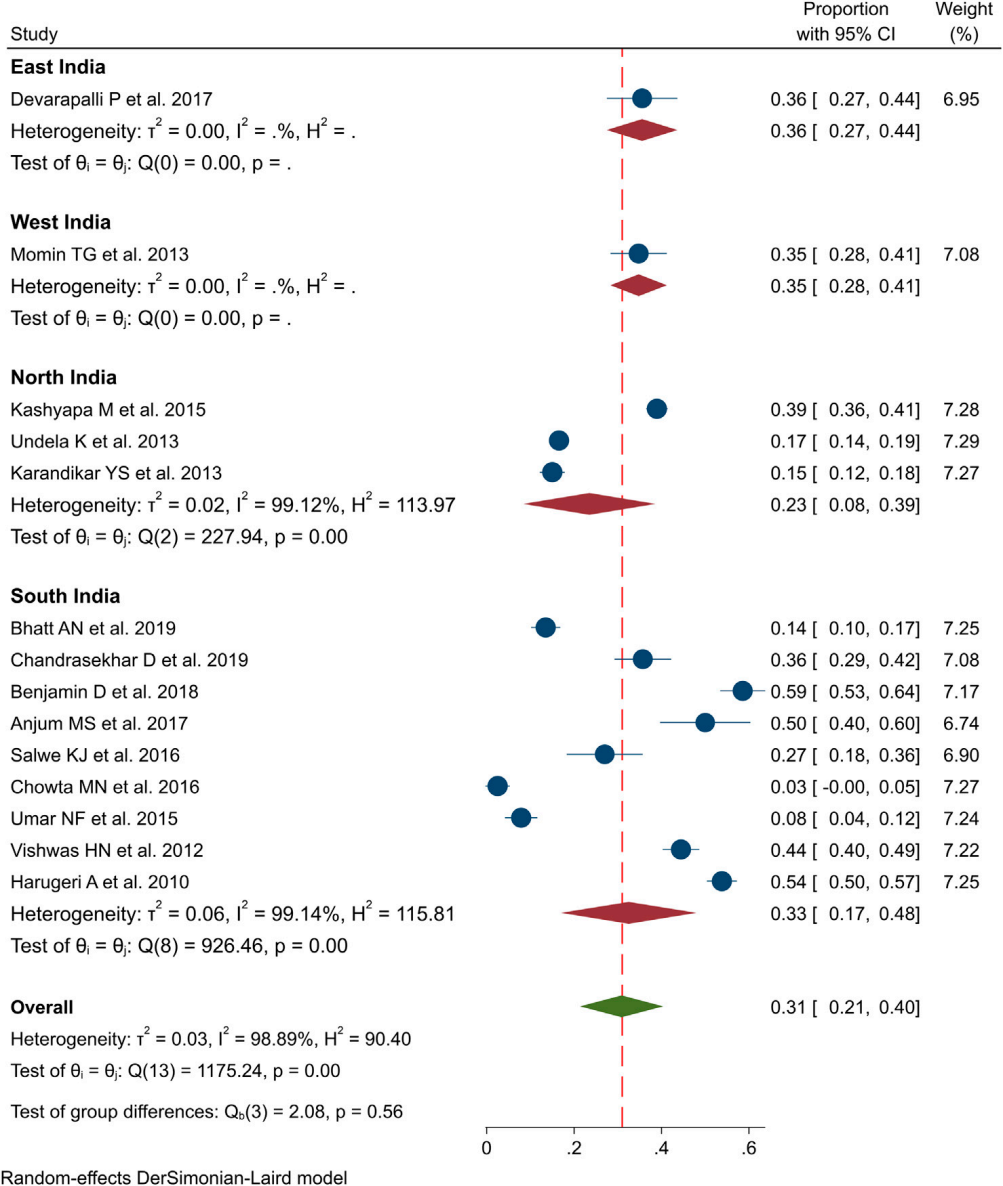
Prevalence of hyperpolypharmacy (≥10 drugs) use in older people across various geographic regions in India.

Fourteen studies investigated the prevalence of hyperpolypharmacy among the older population in India (Bhatt et al., 2019; Chandrasekhar et al., 2019; Benjamin et al., 2018; Devarapalli et al., 2017; Anjum et al., 2017; Salwe et al., 2016; Chowta et al., 2016; Kashyap et al., 2015; Umar et al., 2015; Undela et al., 2014; Karandikar et al., 2013; Momin et al., 2013; Nagendra et al., 2012; Harugeri et al., 2010). The pooled estimate of hyperpolypharmacy was 31% in India (*n* = 6,497, 95% CI: 21–40; *I* ^*2*^ = 98.9%; *p* < 0.01; τ^2^ 0.0321). Region-wize data on the prevalence of hyperpolypharmacy among older adults showed considerable variations with 36% prevalence was seen in East India (95% CI: 27–44), 35% in West India (95% CI: 28–41), 23% in North India (95% CI: 8–39) and 33% (95% CI: 17–48) in South India, as shown in Figure 3. However, these differences between the regions were not statistically significant (*Q* = 2.08, df = 3; *p* = 0.560).

### PIM Use

**FIGURE 4:**
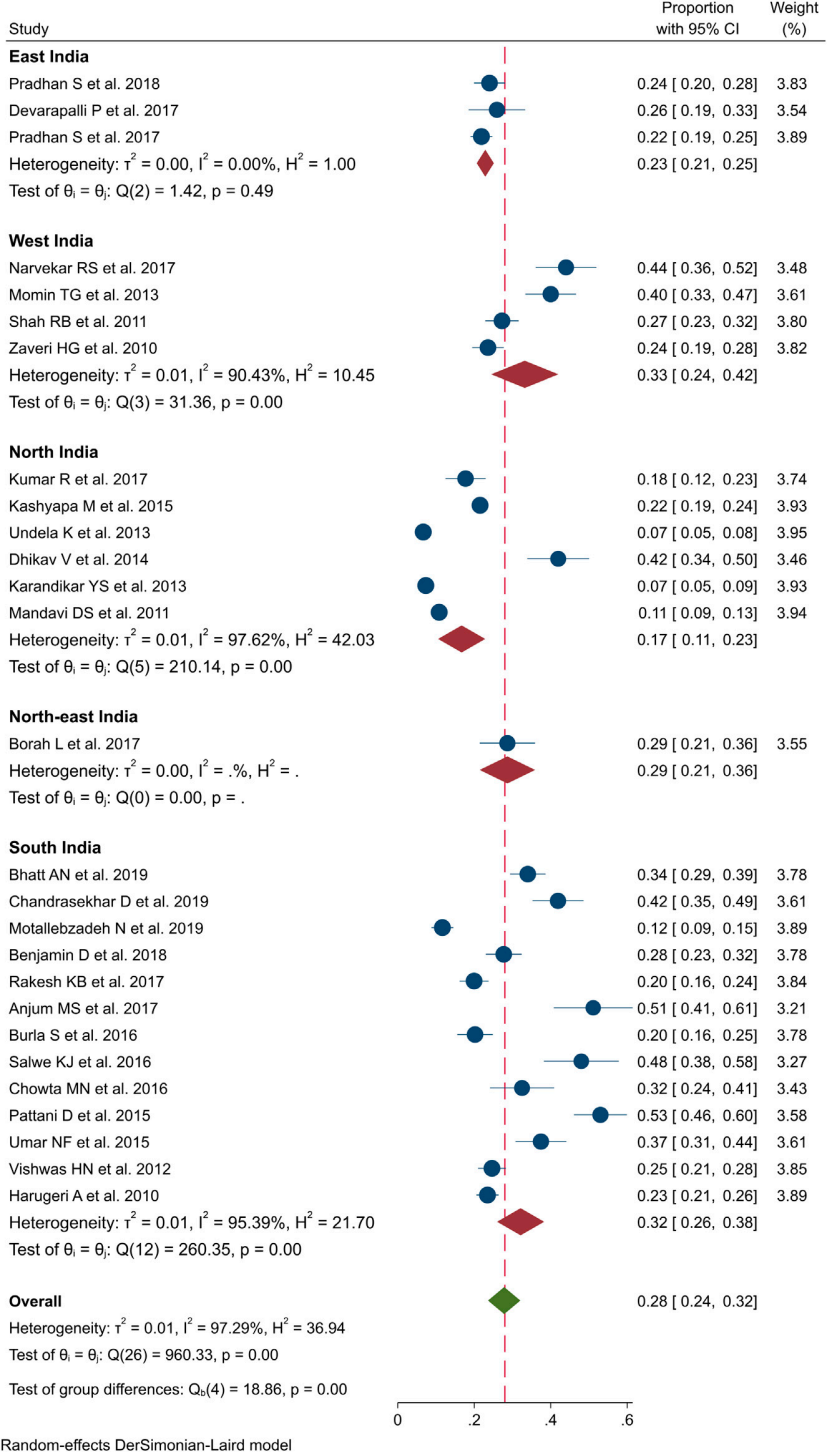
Prevalence of potential inappropriate medication (PIM) use in older people across various geographic regions in India.

All the 27 studies provided PIM estimates among the older population in India (Bhatt et al., 2019; Chandrasekhar et al., 2019; Motallebzadeh et al., 2019; Pradhan and Panda, 2018; Benjamin et al., 2018; Devarapalli et al., 2017; Kumar et al., 2017; Pradhan et al., 2017; Narvekar et al., 2017; Borah et al., 2017; Rakesh et al., 2017; Anjum et al., 2017; Swathi and Bhavika, 2016; Salwe et al., 2016; Chowta et al., 2016; Kashyap et al., 2015; Danisha et al., 2015; Umar et al., 2015; Undela et al., 2014; Dhikav et al., 2014; Karandikar et al., 2013; Momin et al., 2013; Nagendra et al., 2012; Shah et al., 2011; Mandavi et al., 2011; Zaveri et al., 2010; Harugeri et al., 2010). The pooled prevalence of PIM was found to be 28% by using random-effect model (n = 11,649, 95% CI: 24–32; *I* ^*2*^ = 97.3; *p* < 0.01; τ^2^ 0.0117), which indicated substantial heterogeneity, as shown in Figure 4. Comparison of PIM proportions in India showed significant differences among the four regions (NEWS) (*Q* = 18.8, df = 4; *p* < 0.01). West India and South India demonstrated a relatively higher pooled prevalence of 33% (95% CI: 24–42, *p* < 0.01) and 32% (95% CI: 26–38, *p* < 0.01), respectively, while North India and East India had a lower pooled prevalence of 17 and 23%, respectively. The variations in the pooled prevalence of PIM use are further illustrated in the forest plot in Figure 4.

### Stratified Analysis

A stratified meta-analysis of the prevalence of polypharmacy, hyperpolypharmacy, and PIM use in India is summarized in Table 2. We stratified the studies by various baseline characteristics and interrogated the source of heterogeneity and differences between the groups. Significant heterogeneity was detected among all the subgroups; for instance, studies performed for less than 6°months of duration had a higher pooled prevalence of polypharmacy (59%), compared to those conducted for 6–12°months (46%) and >1 year (47%). A significant heterogeneity between the groups was observed (*Q* = 26.4, df = 3; *p* < 0.01). Regarding PIM use, studies conducted before 2013 had a lower pooled prevalence (22%) than those conducted between 2013 and 2016 (31%); however, the pooled prevalence slightly decrease for studies conducted after 2017. Grouping the studies by various subgroups did not reduce heterogeneity, and no significant difference was observed between the groups (duration of the study, mean age, percentage of females, the mean number of medications prescribed, and the number of PIM identified). However, significant differences in the heterogeneity were observed between low-quality and high-quality studies (*Q* = 5.30, df = 1; *p* = 0.021).

**TABLE 2:** Stratified meta-analysis of the prevalence of polypharmacy, hyperpolypharmacy, and potential inappropriate medication (PIM) use in India.

Characteristics	Number of studies	Pooled prevalence in percentage (95% CI)	*p* For interaction^a^	*I* ^*2*^ *(%)*	Z	Heterogeneity between groups		
1. Polypharmacy						*Q*	df	*p*
Year of publication			0.001			0.43	2	0.807
≤2012	3	48 (42–56)		-	7.08			
2013–2016	8	46 (34–58)		98.4	7.57			
≥2017	10	51 (41–61)		97.3	10.1			
Study duration			0.001			26.43	3	<0.001
<6°months	5	59 (47–72)		97.6	9.28			
6–12°months	12	46 (36–55)		97.7	9.51			
>1°year	2	47 (44–50)		-	34.6			
Mean age			0.001			0.41	2	0.81
<70	8	46 (33–59)		99.1	6.90			
≥70	8	50 (42–58)		93.9	12.36			
NA	5	52 (37–66)		97.1	6.96			
Percentage of female			0.001			0.04	1	0.84
<50%	15	49 (41–58)		98.6	11.0			
≥50%	6	48 (38–58)		93.1	9.37			
Average number of drugs			0.001			14.55	2	0.001
<5.5	2	61 (57–65)		-	30.2			
≥5.5	15	49 (41–58)		98.6	11.07			
NA	4	45 (36–54)		88.8	9.78			
Number of PIM use			0.001			0.37	1	0.54
<3	12	47 (41–53)		95.3	14.7			
≥3	9	52 (37–66)		99.0	7.2			
Quality of studies^b^			0.001			0.66	1	0.42
High (≥7)	18	48 (40–54)		98.4	12.36			
Low (<7)	3	56 (38–74)		-	6.01			
2. Hyperpolypharmacy								
Year of publication			0.001			29.81	2	0.001
≤2012	2	50 (47–53)		*-*	37.0			
2013–2016	7	20 (10–31)		*98.7*	3.76			
≥2017	5	39 (19–58)		*98.2*	3.79			
Study duration			0.001			72.9	3	0.001
<6°months	1	27 (19–36)		-	6.08			
6–12°months	10	28 (18–37)		98.6	5.46			
>1°year	1	54 (50–57)		-	30.79			
Mean age			0.001			8.83	2	0.012
<70	6	37 (24–51)		98.8	5.55			
≥70	6	24 (8–40)		98.8	2.99			
Na	2	17 (15–20)		-	12.45			
Percentage of female			0.001			0.82	1	0.365
<50%	10	33 (22–44)		98.8	5.99			
≥50%	4	25 (9–40)		97.9	3.07			
Average number of drugs			0.001					0.001
<5.5	1	3 (1–7)		-	1.75			
≥5.5	10	36 (26–46)		98.6	6.82			
Na	1	23 (8–37)		-	3.06			
Number of PIM use			0.001			0.70	1	0.403
<3	9	34 (21–47)		98.85	5.12			
≥3	5	25 (9–41)		98.12	3.08			
Quality of studies^b^			0.001			31.2	1	0.001
High (≥7)	12	34 (24–43)		*98.7*	6.76			
Low (<7)	2	5 (2–5)		*-*	3.53			
3. PIM use								
Year of publication			0.449			3.33	2	0.189
≤2012	5	22 (15–29)		93.9	5.87			
2013–2016	10	31 (24–39)		97.3	8.17			
≥2017	12	28 (23–34)		96.3	10.04			
Study duration			0.930			7.46	3	0.059
<6°months	7	27 (19–34)		96.2	7.0			
6–12°months	15	31 (24–37)		97.0	9.21			
>1°year	3	23 (20–27)		-	12.41			
Mean age			0.072			5.76	2	0.056
<70	9	24 (19–30)		96.7	8.64			
≥70	9	35 (28–42)		93.9	9.66			
Na	9	25 (19–32)		94.5	7.68			
Percentage of female			0.179			1.57	1	0.210
<50%	20	26 (22–30)		96.0	12.92			
≥50%	7	34 (22–46)		96.3	5.74			
Average number of drugs			0.548			4.08	2	0.130
<5.5	4	23 (18–27)		70.5	9.54			
≥5.5	17	27 (22–31)		96.1	12.11			
Na	6	35 (22–48)		97.5	5.31			
Number of PIM use			0.782			0.15	1	0.702
<3	17	27 (23–32)		95.7	11.57			
≥3	10	29 (22–36)		96.8	8.24			
Quality of studies^b^			0.112			5.30	1	0.021
High (≥7)	23	27 (23–30)		96.2	13.50			
Low (<7)	4	37 (29–46)		75.8	8.68			

a *p*-value from meta-regression analyses,

b New-Castle Ottawa scale score, PIM: potential inappropriate medication.

### Subgroup Analysis

Subgroup analysis by geographic region, study design, type of hospital, and study settings did not influence the prevalence estimates of polypharmacy, hyperpolypharmacy, and PIM use, as shown in Table 3. However, the prevalence of hyperpolypharmacy in outpatient settings (8%) and cross-sectional studies (14%) was low. The prevalence of PIM use varied between inpatient (31%) and outpatient settings (25%); however, lower prevalence of PIM use was reported in government hospitals (25%).

**TABLE 3:** Subgroup analysis for the potential variables between studies of prevalence of polypharmacy, hyperpolypharmacy and potential inappropriate medication (PIM) use in older population in India.

Subgroups		No of studies	Prevalence (95%CI)	Test for heterogeneity			Between subgroup differences		
				*Tau* ^*2*^	*p*	*I* ^*2*^	*Q*	*df*	*p*
Polypharmacy									
Geographical region	South India	12	49% (42–57%)	0.0148	<0.01	99%	28.25	4	<0.001
	East India	3	52% (27–77%)	0.0472	<0.01	99%			
	North India	4	39% (22–56%)	0.0298	<0/01	99%			
	West India	1	51% (44–58%)	-	-	-			
	North east India	1	72% (65–79%)	-	-	-			
Study design	Cross-sectional	8	55% (44–65%)	0.0234	<0.01	97%	1.70	1	0.191
	Cohort	13	45% (37–54%)	0.0242	<0.01	98%			
Hospital	Government	8	53% (38–67%)	0.0428	<0.01	99%	0.65	1	0.418
	Private	13	46% (41–52%)	0.0102	<0.01	93%			
Setting	Inpatient	12	43% (35–51%)	0.0168	<0.01	97%	5.11	2	0.077
	Outpatient	7	57% (47–67%)	0.0169	<0.01	97%			
	Both in-and-outpatient	2	56% (26–87%)	0.0472	<0.01	98%			
Hyper polypharmacy									
Geographical region	South India	9	33% (17–48%)	0.0551	<0.01	99%	2.08	3	0.555
	East India	1	36% (27–44%)	-	-	-			
	North India	3	23% (8–39%)	0.0176	<0.01	99%			
	West India	1	35% (28–41%)	-	-	-			
Study design	Cross-sectional	4	14% (6–22%)	0.0059	<0.01	95%	11.65	1	<0.001
	Cohort	10	38% (26–49%)	0.0313	<0.01	99%			
Hospital	Government	3	30% (13–47%)	0.0218	<0.01	99%	0.01	1	0.916
	Private	11	31% (19–44%)	0.0444	<0.01	99%			
Setting	Inpatient	11	37% (26–47%)	0.0305	<0.01	99%	17.44	2	<0.001
	Outpatient	2	8% (0–19%)	0.0058	<0.01	96%			
	Both in-and-outpatient	1	15% (12–18%)	-	-	-			
PIM use									
Geographical region	South India	13	32% (26–38%)	0.0118	<0.01	95%	18.86	4	0.001
	East India	3	23% (21–25%)	0	0.49	0%			
	North India	6	17% (11–23%)	0.0055	<0.01	98%			
	West India	4	33% (24–42%)	0.0071	<0.01	90%			
	North east India	1	29% (21–36%)	-	-	-			
Study design	Cross-sectional	8	27% (18–35%)	0.0127	<0.01	97%	0.16	1	0.693
	Cohort	19	28% (23–34%)	0.0127	<0.01	98%			
Hospital	Government	13	25% (19–30%)	0.0095	<0.01	97%	1.82	1	0.176
	Private	14	31% (24–38%)	0.0166	<0.01	97%			
Setting	Inpatient	15	31% (24–38%)	0.0169	<0.01	98%	2.47	2	0.290
	Outpatient	9	25% (19–31%)	0.0075	<0.01	95%			
	Both in-and-outpatient	3	21% (5–37%)	0.0190	<0.01	98%			

### Publication Bias Assessment

The Egger’s and Begg’s tests indicated statistically significant publication bias for the polypharmacy estimates (Egger test: *p* = 0.034) and PIM use (Egger test: *p* = 0.027 & Begg’s test: *p* = 0.001). Visual examination of the funnel plots showed asymmetry and suggested publication bias, as shown in Supplementary Figure S1.

## Discussion

Overuse and misuse of medications in the older population are among the major concerns in India (Porter and Grills, 2016). The growing culture of irrational and unnecessary prescribing of medications in the older population may increase the risk of adverse outcomes. Multiple studies demonstrated that poor prescribing practices (Chaturvedi et al., 2012), inappropriate medication selection (Boralkar et al., 2011; Castelino et al., 2011), and frequent misuse of drugs to earn profits (Roy et al., 2007; Kotwani et al., 2010) are some of the factors that result in polypharmacy, hyperpolypharmacy, and PIM use in India. In particular, older people with multiple comorbidities are exposed to polypharmacy, and suboptimal prescribing may increase their likelihood of receiving PIMs (Fabbietti et al., 2018).

We assessed the prevalence of polypharmacy, hyperpolypharmacy, and PIM use among the older population through a comprehensive systematic review and reported regional differences in prevalence across four regions in India. Data from 27 studies (11,649 participants) reported a higher prevalence of polypharmacy (49%), hyperpolypharmacy (31%), and PIM use (28%) among the older population in India. Region-specific estimates showed that polypharmacy is widely prevalent in Northern India (72%), hyperpolypharmacy in the eastern and western parts of India (36%), and PIM use (33%) in Western states. Furthermore, polypharmacy is more frequently observed in outpatient settings (57%) and hyperpolypharmacy in inpatient settings (37%). Stratified analysis showed variations in PIM exposure across subsets, and governmental hospitals showed a lower prevalence of PIM use than private hospitals (25 vs. 27%). Considerable variations in polypharmacy and hyperpolypharmacy are seen among cross-sectional studies in comparison to cohort studies.

The regional differences in the prevalence of polypharmacy, hyperpolypharmacy, and PIM use among the older population noted in our study could be due to the inclusion of a limited number of studies, smaller sample size, and differences in socioeconomic conditions, risk factors, and quality of healthcare services across the four regions of India. Two-thirds of the studies were conducted in South India, where the level of awareness of geriatric care and polypharmacy prevalence was very high compared to other regions. The *Sharma et al.* study demonstrated the differences in the prevalence of polypharmacy in the Indian states and reported that Uttaranchal (93.1%) from North India and Southern states, such as Telangana (82.8%) and Karnataka (84.6%), had the highest prevalence of polypharmacy compared to Northeast -West Bengal (5.8%), Tripura (East India) (6.8%), Madhya Pradesh (central India) (8.3%) and Goa (West India) (13.8%) (Pravodelov, 2020). While the underlying reasons for the increasing prevalence of polypharmacy are still unknown, our findings highlight the need to develop strategies to reduce polypharmacy in clinical practice and motivate physicians to adopt more judicious prescribing to reduce the number of medications among the older population in India.

Polypharmacy and hyperpolypharmacy are proxy indicators for PIM use in older populations, leading to adverse clinical outcomes. The findings of the current study revealed an increasing incidence of hyperpolypharmacy. The pooled estimates showed a much higher prevalence of hyperpolypharmacy (31%) in India than the developed countries like the United States of America (1%) (Assari et al., 2019), New Zealand (2.1%) (Nishtala and Salahudeen, 2015), Australia (8%) (Wylie et al., 2020), Sweden (18%) (Hovstadius et al., 2010), and Finland (28%) (Jyrkkä et al., 2009). Several review articles suggest that the application of clinical guidelines in the older population may contribute to hyperpolypharmacy (Hilmer and Gazarian, 2008; Scott and Guyatt, 2010; Kojima et al., 2020). However, it is widely recognized that the evidence-based guidelines are derived from clinical trials that generally exclude older patients with comorbidity (Sheridan and Julian, 2016; Guthrie and Boyd, 2018). This research provides vital information to alert clinicians and researchers about the dire need to reduce the medication burden in older people.

Our findings on the pooled prevalence of PIM use showed that 28% of older patients in India are affected by PIM; a similar trend was observed over the years in high-income countries (33.3%) (Liew et al., 2019). This study did not identify potential variations across different regions ranging from 33 to 36%, except North India (23%). In a recent meta-analysis of studies conducted among older patients in primary care settings, the pooled prevalence of PIM use was 33.3% in high-income countries, varying from 24.8% in North America to 59.2% in Australasians (Liew et al., 2019). In the same study, the prevalence of PIM use in middle-income countries was 23.2%. With the increase in the older population, our pooled results suggest a need for multi-pronged approaches to address PIM use in India. Some approaches include medication reviews by clinical pharmacists and the implementation of a computerized clinical decision support system. Moreover, there is a need to plan broader interprofessional interventions to motivate clinicians to reduce polypharmacy and improve the optimal use of medications in older people. The World Health Organization suggested monitoring and rectifying potential PIM prescribing regularly and prioritizing medication safety at the national level to reduce PIM use in the older population (World Health Organization, 2017).

Findings from this study demonstrate the prevalence of polypharmacy, hyperpolypharmacy, and PIM use in Indian states and highlight the urgency to address inappropriate medication use in the older population. Therefore, future studies with a multi-pronged approach should be conducted, focusing on comprehensive geriatric medication reviews by clinical pharmacists, computerized clinical decision support systems, and prioritizing rational geriatric prescribing at the national level. Moreover, multifaceted randomized controlled trials are needed to evaluate the effects of the intervention on clinically relevant outcomes such as hospitalization, medication costs, and health-related quality of life.

### Strengths and Limitations

This is the first systematic review and meta-analyses to consolidate the quantitative evidence on the wide-ranging impact of polypharmacy, hyperpolypharmacy, PIM use in various states of India. We also thoroughly assessed the risk of bias in each of the 27 observational studies. We further conducted meta-analyses stratified according to the suspected potential source of heterogeneity between the studies and subgroups.

The study findings are subject to some limitations. First, factors like geographic areas, cultures, and practices vary widely across the states in India, which may influence the results. Second, higher heterogeneity in the outcomes may be due to differences in sample size (ranging from a few hundred to a few thousand). Low power and precision may produce higher Cochran Q (heterogeneity x^2^ test statistics) and *I* ^*2*^. More studies were conducted in South India than in any other region. Third, publication bias was present in the selected studies and has been known to affect heterogeneity. We performed a more stratified subgroup analysis to explore the source of heterogeneity and differences within the subsets.

## Conclusion

The prevalence of polypharmacy and hyperpolypharmacy among older Indian adults is relatively high. Almost a quarter of the older people are affected by PIM use in India. Significant regional differences exist in the prevalence of polypharmacy, hyperpolypharmacy, and PIM use. These findings highlight the need for urgent steps to promote rational geriatric prescribing and prioritize pharmacist-led comprehensive medication reviews to reduce medication-related problems among older people in India.

## Data Availability

The original contributions presented in the study are included in the article/Supplementary Material, further inquiries can be directed to the corresponding author.
