# Supplementary material for: Prevalence of Polypharmacy, Hyperpolypharmacy and Potentially Inappropriate Medication Use in Older Adults in India: A Systematic Review and Meta-Analysis
Source: Front Pharmacol. 2021 May 19;12:685518. doi: 10.3389/fphar.2021.685518 (PMC8173298; doi:10.3389/fphar.2021.685518)
Supplement: Supplementary file 1 [file DataSheet1.pdf]

Table 1: Search strategy

PubMed/MEDLINE	Sciencedirect/Scopus	Embase	Google scholar
"polypharmacy"[MeSH Terms] OR "polypharmacy"[All Fields]"beers criteria"[All Fields] AND "potential inappropriate medication"[All Fields] AND "india"[All Fields] AND "stopp 2 criteria"[All Fields] AND "stopp criteria"[All Fields] "hyperpolypharmacy"[All Fields] potentially inappropriate medication list"[All Fields] inappropriate medication, Beers, STOPP, inappropriate, medication, drug, use, prescribing, and elderlyTS=("Potentially Inappropriate Medication List" OR “PIM List*” OR “Inappropriate Medication*” OR “Inappropriate Medicine*” OR “Inappropriate Prescrib*” OR “Inappropriate Prescription*” OR “Inappropriate Drug*” OR “Suboptimal Medication*” OR “Suboptimal Medicine” OR “Suboptimal Medicines” OR	“potentially inappropriate prescription*” OR “potentially inappropriate medication*” OR “potentially inappropriate medicine*” OR “potentially inappropriate drug*” OR “Beers Criteria” OR STOPP OR “Screening Tool of Older Person's Potentially Inappropriate Prescriptions” OR “PIM”) "hyperpolypharmacy"[All Fields] TS=("Polypharmacy" OR "Polymedication*" OR "Polymedicine" OR "Polyprescription*" OR "Polydrug*" OR "Multiple Prescription*" OR "Multiple Prescrib*" OR "Multiple Medication*" OR "Multiple Medicine" OR "Multiple Medicines" OR "Multiple Drug*" OR "Multiple Drug Use" OR "Geriatric assessment" OR "Geriatric assessments" OR "Geriatric review" OR "Geriatric reviews") india AND 'inappropriate prescribing'	"hyperpolypharmacy"[All Fields] 'potential inappropriate medication' OR (('potential'/exp OR potential) AND inappropriate AND ('medication'/exp OR medication)) 'beers criteria'/exp OR 'beers criteria' 'inappropriate prescribing'/exp OR 'inappropriate prescribing' 'polypharmacy'/exp OR polypharmacy 'stopp criteria'/exp OR 'stopp criteria' 'older adults' AND 'inappropriate prescribing' india AND 'inappropriate prescribing'	potentially inappropriate medication OR prescribing OR Beers, STOPP, inappropriate, medication OR drug use“Beers Criteria” OR STOPP OR “Screening Tool of Older Person's Potentially Inappropriate Prescriptions” OR “PIM”) Polypharmacy" OR "Polymedication*" OR "Polymedicine" OR "Polyprescription" OR "hyperpolypharmacy"[All Fields] OR "Polydrug*" OR "Multiple Prescription*" india AND 'inappropriate prescribing'

“Suboptimal Prescrib*” OR “Suboptimal Prescription*” OR “Suboptimal Drug Use” OR “Suboptimal Drugs” OR “Incorrect Medication*” OR “Incorrect Prescribing” OR “Incorrect Prescription*” OR “Incorrect Drug*” OR “potentially inappropriate prescrib*”.<br>india AND 'inappropriate prescribing'			

Suplementary Table 2: Excluded articles with reason

No	Authors	Title	Year	Reason for exclusion
1	Tejus A., Saxena S.K., Dwivedi A.K., Salmani M.F., Pradhan S.	Analysis of the prescription pattern of psychotropics in an outpatient department of a general hospital psychiatry unit	2020	Conference abstract
2	Asiri S., Mahmoudi F., Pasha A., Leyli E.K.	Drug use pattern among elderly people referred to the retirement centers	2018	Conference abstract
3	Al-Qerem W., Jarrar Y.B., Al-Sheikh I., Elmaadani A.	The prevalence of drug-drug interactions and polypharmacy among elderly patients in Jordan	2018	Conference abstract
4	Nishandar T.B., Kale A.S., Pise H.N.	Drug utilization pattern in geriatric patients attending outpatient department at rural tertiary care hospital in Maharashtra	2017	Conference abstract
5	Joseph J., Ramesh M., Harugeri A., Parthasarathi G., Basavanagowdappa H.	Frequency and nature of medication-related problems in elderly Indian inpatients	2016	Conference abstract
6	Kolhe A., Kale A.S., Padwal S.L.	Drug utilization study in geriatric patients at rural tertiary care hospital	2015	Conference abstract
7	Hummel J.	Ambulatory Care for Geriatric Patients [Ambulante Versorgung geriatrischer Patienten: Altersmedizin]	2020	Editorial
8	Tetteh E.K.	Reducing avoidable medication-related harm: What will it take?	2019	Editorial
9	Salhotra R., Tyagi A.	Medication errors: They continue	2019	Editorial
10	Olaniyan J.O., Ghaleb M., Dhillon S., Robinson P.	Safety of medication use in primary care	2015	Editorial
11	Saqlain M., Ahmed Z., Butt S.A., Khan A., Ahmed A., Ali H.	Prevalence of potentially inappropriate medications use and associated risk factors among elderly cardiac patients using the 2015 American Geriatrics Society beers criteria	2020	Population irrelevant
12	Heydari M., Mehraeen M., Joulaei H.	Over medication and waste of resources in physicians' prescriptions: A cross sectional study in Southwestern Iran	2020	Population irrelevant
13	Tefera G.M., Feyisa B.B., Umeta G.T., Kebede T.M.	Predictors of prolonged length of hospital stay and in-hospital mortality among adult patients admitted at the surgical ward of Jimma University medical center, Ethiopia: Prospective observational study	2020	Population irrelevant
14	Thinuan P., Siviroj P., Lerttrakarnnon P., Lorga T.	Prevalence and potential predictors of frailty among community-dwelling older persons in Northern Thailand: A cross-sectional study	2020	Population irrelevant

15	Mishore K.M., Girma Y., Tola A., Mekuria A.N., Ayele Y.	Evaluation of Medication Use Pattern Among Patients Presenting to the Emergency Department of Hiwot Fana Specialized University Hospital, Using WHO Prescribing Indicators	2020	Population irrelevant
16	Kucukdagli P., Bahat G., Bay I., Kilic C., Oren M.M., Turkmen B.O., Karan M.A.	The relationship between common geriatric syndromes and potentially inappropriate medication use among older adults	2020	Population irrelevant
17	Saqlain M., Ali H., Kamran S., Munir M.U., Jahan S., Mazhar F.	Potentially inappropriate medications use and its association with health-related quality of life among elderly cardiac patients	2020	Population irrelevant
18	Nagai T., Wakabayashi H., Maeda K., Momosaki R., Nishiyama A., Murata H., Uei H.	Influence of potentially inappropriate medications on activities of daily living for patients with osteoporotic vertebral compression fractures: A retrospective cohort study	2020	Population irrelevant
19	Bose N.M., Mathew M., Joy B., Joel J.J., Ks R., Raj B., Nandakumar U.P., Vijayan A.	Assessment of drug related problems in the general medicine ward of a multispeciality teaching hospital	2019	Population irrelevant
20	Albaghdadi O., Morteza M.H., Rahi F.A.	Potentially inappropriate medications use in a population of iraqi geriatric outpatients according to beers criteria	2019	Population irrelevant
21	Saboor M., Momtaz Y.A., Kamrani A.-A.A., Sahaf R.	Prescription pattern among Iranian community dwelling older adults	2019	Population irrelevant
22	Kaplan Ç., Bılge U., Acar N., Ünlüoğlu İ.	Determination of polypharmacy and inappropriate drug use in patients aged over 65 years who presented to the emergency department [Acıl servıse başvuran 65 yaş üzerı hastalarda polıfarması ve uygunsuz ılaç kullaniminin belırlenmesı]	2019	Population irrelevant
23	Chang C.-B., Lai H.-Y., Hwang S.-J., Yang S.-Y., Wu R.-S., Liu H.-C., Chan D.-C.	Prescription of potentially inappropriate medication to older patients presenting to the emergency department: a nationally representative population study	2018	Population irrelevant
24	Prasert V., Akazawa M., Shono A., Chanjaruporn F., Ploylearmsang C., Muangyim K., Wattanapongsatit T., Sutin U.	Applying the Lists of Risk Drugs for Thai Elderly (LRDTE) as a mechanism to account for patient age and medicine severity in assessing potentially inappropriate medication use	2018	Population irrelevant
25	Abegaz T.M., Birru E.M., Mekonnen G.B.	Potentially inappropriate prescribing in Ethiopian geriatric patients hospitalized with cardiovascular disorders using START/STOPP criteria	2018	Population irrelevant
26	Biswas A., Sinha N., Ray K., Tripathi S.K.	A study on drug use and medication management perspectives among elderly and the impact of professional oversight	2018	Population irrelevant

27	Yilmaz F., Colak M.Y.	Evaluation of inappropriate medication use and compliance in elderly people	2018	Population irrelevant
28	Abdulah R., Insani W.N., Destiani D.P., Rohmaniasari N., Mohenathas N.D., Barliana M.I.	Polypharmacy leads to increased prevalence of potentially inappropriate medication in the indonesian geriatric population visiting primary care facilities	2018	Population irrelevant
29	Ozalas S.M., Huang V., Brunetti L., Reilly T.	Comparison of two versions of the beers criteria and adverse outcomes in older hospitalized patients	2017	Population irrelevant
30	Bahat G., Bay I., Tufan A., Tufan F., Kilic C., Karan M.A.	Prevalence of potentially inappropriate prescribing among older adults: A comparison of the Beers 2012 and Screening Tool of Older Person's Prescriptions criteria version 2	2017	Population irrelevant
31	Rathod Mrudangsinh M., Achankunju A., John R.M., Sam G.K.	A study to review the appropriateness of drug dosage in renally impaired patient by identifying, analysing, and adjusting dosages in a tertiary care teaching hospital	2017	Population irrelevant
32	Lim L.M., McStea M., Chung W.W., Azmi N.N., Aziz S.A.A., Alwi S., Kamarulzaman A., Kamaruzzaman S.B., Chua S.S., Rajasuriar R.	Prevalence, risk factors and health outcomes associated with polypharmacy among urban community-dwelling older adults in multiethnic Malaysia	2017	Population irrelevant
33	Gencer M.Z., Arica S.	Use of polypharmacy and herbal medication on quality of life in elderly patients at okmeydani hospital’s polyclinics in Istanbul, Turkey	2017	Population irrelevant
34	Al-Hashar A., Al Sinawi H., Al Mahrizi A., Al-Hatrushi M.	Prevalence and covariates of polypharmacy in elderly patients on discharge from a tertiary care hospital in Oman	2016	Population irrelevant
35	Pradhan S., Panda A., Panigrahy S.R.	Analysis of drug utilization pattern in elderly in an outpatient department using who indicators: A cross sectional study	2016	Population irrelevant
36	Ramanath K.V., Chaudhary S.	Study on pharmaceutical care in geriatrics of a rural tertiary care hospital	2016	Population irrelevant
37	Hudhra K., García-Caballos M., Casado-Fernandez E., Jucja B., Shabani D., Bueno-Cavanillas A.	Polypharmacy and potentially inappropriate prescriptions identified by Beers and STOPP criteria in co-morbid older patients at hospital discharge	2016	Population irrelevant
38	Cullinan S., O'Mahony D., O'Sullivan D., Byrne S.	Use of a frailty index to identify potentially inappropriate prescribing and adverse drug reaction risks in older patients	2016	Population irrelevant
39	Prasanth N.V., Vinod B.	A prospective surveillance study on the prevalence and influential determinants of polypharmacy in hospitalized geriatric patients	2016	Population irrelevant

40	Fadare J.O., Desalu O.O., Obimakinde A.M., Adeoti A.O., Agboola S.M., Aina F.O.	Prevalence of inappropriate medication prescription in the elderly in Nigeria: A comparison of Beers and STOPP criteria	2015	Population irrelevant
41	Chou C.-L., Perng D.-W., Lin T.-L., Lin A.M.-Y., Chen T.-J., Wu M.-S., Chou Y.-C.	Analysis of Prescription Pattern and Guideline Adherence in the Management of Asthma among Medical Institutions and Physician Specialties in Taiwan between 2000 and 2010	2015	Population irrelevant
42	Ahmad A., Khan M.U., Haque I., Ivan R., Dasari R., Revanker M., Pravina A., Kuriakose S.	Evaluation of potential drug - drug interactions in general medicine ward of teaching hospital in Southern India	2015	Population irrelevant
43	Chang C.-B., Yang S.-Y., Lai H.-Y., Wu R.-S., Liu H.-C., Hsu H.-Y., Hwang S.-J., Chan D.-C.	Application of three different sets of explicit criteria for assessing inappropriate prescribing in older patients: A nationwide prevalence study of ambulatory care visits in Taiwan	2015	Population irrelevant
44	Abraham F., Varughese G., Mathew J.C., John P.M., Sam G.K.	Drug utilization pattern among geriatric patients in a tertiary care teaching Hospital	2015	Population irrelevant
45	Bhad R., Hazari N.	Combining pharmacological treatments in geriatric population: Weighing the balance	2015	Population irrelevant
46	Rasu R.S., Iqbal M., Hanifi S.M.A., Moula A., Hoque S., Rasheed S., Bhuiya A.	Level, pattern, and determinants of polypharmacy and inappropriate use of medications by village doctors in a rural area of Bangladesh	2014	Population irrelevant
47	Hien H., Berthé A., Drabo M.K., Meda N., Konaté B., Tou F., Badini-Kinda F., Macq J.	Prevalence and patterns of multimorbidity among the elderly in Burkina Faso: Cross-sectional study	2014	Population irrelevant
48	Clares J.W.B., De Freitas M.C., Borges C.L.	Social and clinical factors causing mobility limitations in the elderly [Fatores sociais e clínicos que causam limitac¸ão da mobilidade de idosos]	2014	Population irrelevant
49	Jhaveri B., Patel T., Barvaliya M., Tripathi C.B.	Drug utilization pattern and pharmacoeconomic analysis in geriatric medical in-patients of a tertiary care hospital of India	2014	Population irrelevant
50	Zyoud S.H., Abd-Alhafez A.B., Hussein A.O., Abu-Shehab I.S., Al-Jabi S.W., Sweileh W.M.	Patterns of use of medications, herbal products and nutritional supplements and polypharmacy associating factors in Palestinian geriatric patients	2014	Population irrelevant
51	Shah V.N.	Drug utilization pattern and pharmacoeconomic analysis in geriatric medical in-patients of a tertiary care hospital of India	2014	Population irrelevant
52	Thakkar K.B., Jain M.M., Billa G., Joshi A., Khobragade A.A.	A drug utilization study of psychotropic drugs prescribed in the psychiatry outpatient department of a tertiary care hospital	2013	Population irrelevant

53	Eng M.L., Birch J.T., Jr.	Medication use and polypharmacy in older adults	2013	Population irrelevant
54	Fadare J.O., Agboola S.M., Opeke O.A., Alabi R.A.	Prescription pattern and prevalence of potentially inappropriate medications among elderly patients in a Nigerian rural tertiary hospital	2013	Population irrelevant
55	Dhamija P., Bansal D., Srinivasan A., Bhalla A., Hota D., Chakrabarti A.	Patterns of prescription drug use and incidence of drug-drug interactions in patients reporting to medical emergency	2013	Population irrelevant
56	Abreu M.S., Ferreira S.D.A., Ferreira L.P.L., Toneo Júnior J.F., Maciel W.V., Maciel S.S.S.V.	Prevalence and costs of hospitalizations for poisoning and accidental intoxication in Brazilian elderly	2013	Population irrelevant
57	Wu C., Bell C.M., Wodchis W.P.	Incidence and economic burden of adverse drug reactions among elderly patients in Ontario emergency departments: A retrospective study	2012	Population irrelevant
58	Romana A., Kamath L., Sarda A., Muraraiah S., Jayanthi C.R.	Polypharmacy leading to adverse drug reactions in elderly in a tertiary care hospital	2012	Population irrelevant
59	Yong T.Y., Lau S.Y., Li J.Y., Hakendorf P., Thompson C.H.	Medication prescription among elderly patients admitted through an acute assessment unit	2012	Population irrelevant
60	Grover S., Kumar V., Avasthi A., Kulhara P.	First prescription of new elderly patients attending the psychiatry outpatient of a tertiary care institute in North India	2012	Population irrelevant
61	Rambhade S., Chakarborty A., Shrivastava A., Patil U.K., Rambhade A.	A survey on polypharmacy and use of inappropriate medications	2012	Population irrelevant
62	Ramanath K.V., Nedumballi S.	Assessment of medication-related problems in geriatric patients of a rural tertiary care hospital	2012	Population irrelevant
63	Pudasaini N., Singh C., Sagar G.C., Sapkota S.	Drug prescribing pattern and prescription error in elderly: A retrospective study of inpatient record	2011	Population irrelevant
64	Arvind Nag K., Umesh M., Churi S.	Assessment of drug-drug interactions in hospitalised patients in India	2011	Population irrelevant
65	Pudasaini N., Singh C., Gc S., Sujata S.	Drug prescribing pattern and prescription error in elderly: A retrospective study of inpatient record	2011	Population irrelevant
66	Ünsal A., Demir G.	The prevalence of chronic disease and drug use in the elderly in central Kirşehir	2010	Population irrelevant
67	Harugeri A., Joseph J., Parthasarathi G., Ramesh M., Guido S.	Prescribing patterns and predictors of high-level polypharmacy in the elderly population: A prospective surveillance study from two teaching hospitals in india	2010	Population irrelevant

68	Joseph J., Ramesh M., Harugeri A., Parthasarathi G., Basavanagowdappa H.	Frequency and nature of medication-related problems in elderly Indian inpatients	2010	Population irrelevant
69	Pote S., Tiwari P., D'Cruz S.	Medication prescribing errors in a public teaching hospital in India: A prospective study	2007	Population irrelevant
70	Alam K., Mishra P., Prabhu M., Shankar P.R., Palaian S., Bhandari R.B., Bista D.	A study on rational drug prescribing and dispensing in outpatients in a tertiary care teaching hospital of Western Nepal	2006	Population irrelevant
71	Mandavi, Tiwari P.	Profile of pharmacotherapy in elderly Indian patients: Preliminary findings	2006	Population irrelevant
72	Rehan H.S., Singh C., Tripathi C.D., Kela A.K.	Study of drug utilization pattern in dental OPD at tertiary care teaching hospital.	2001	Population irrelevant
73	St. Louis E.K.	Truly "rational" polytherapy: Maximizing efficacy and minimizing drug interactions, drug load, and adverse effects	2009	Population irrelevant
74	Banning M.	A review of interventions used to improve adherence to medication in older people	2009	Population irrelevant
75	Sonal Sekhar M., Adheena Mary C., Anju P.G., Hamsa N.A.	Study on drug related hospital admissions in a tertiary care hospital in South India	2011	Population irrelevant
76	Sharma R., Arora M., Garg R., Bansal P.	A closer look at the 2019 Beers criteria	2020	Review
77	Karlsson S., Ridbäck A., Brobeck E., Norell Pejner M.	Health Promotion Practices in Nursing for Elderly Persons in Municipal Home Care: An Integrative Literature Review	2020	Review
78	Khan N., Chattopadhyay K., Leonardi-Bee J.	Incidence, prevalence, risk factors and health consequences of polypharmacy in adults in South Asia: A systematic review protocol	2019	Review
79	Shitu Z., Aung M.M.T., Kamauzaman T.H.T., Bhagat V., Rahman A.F.A.	Medication error in hospitals and effective intervention strategies: A systematic review	2019	Review
80	Verma A., Aggarwal S., Garg S., Anand P.	Geriatric health care in India: A review	2019	Review
81	Baclet N., Ficheur G., Alfandari S., Ferret L., Senneville E., Chazard E., Beuscart J.-B.	Explicit definitions of potentially inappropriate prescriptions of antibiotics in older patients: a compilation derived from a systematic review	2017	Review

82	Mohamadloo A., Ramezankhani A., Zarein-Dolab S., Salamzadeh J., Mohamadloo F.	A systematic review of main factors leading to irrational prescription of medicine	2017	Review
83	Patel T.K., Patel P.B.	Incidence of adverse drug reactions in Indian hospitals: A systematic review of prospective studies	2016	Review
84	Nagaratnam N., Nagaratnam K., Cheuk G.	Diseases in the elderly: Age-related changes and pathophysiology	2016	Review
85	Porter G., Grills N.	Medication misuse in India: A major public health issue in India	2016	Review
86	Nagaraju K., Manasa S., Manjunath R.	Pharmacovigilance study in geriatric population	2015	Review
87	Nivya K., Sri Sai Kiran V., Ragoo N., Jayaprakash B., Sonal Sekhar M.	Systemic review on drug related hospital admissions - A pubmed based search	2015	Review
88	Rubio-Valera M., Chen T.F., O'Reilly C.L.	New Roles for Pharmacists in Community Mental Health Care: A Narrative Review	2014	Review
89	Skoog J., Midlöv P., Beckman A., Sundquist J., Halling A.	Drugs prescribed by general practitioners according to age, gender and socioeconomic status after adjustment for multimorbidity level	2014	Review
90	Gupta M., Agarwal M.	Understanding medication errors in the elderly	2013	Review
91	Jose J.	Promoting drug safety in elderly - Needs a proactive approach	2012	Review
92	Sergi G., De Rui M., Sarti S., Manzato E.	Polypharmacy in the elderly: Can comprehensive geriatric assessment reduce inappropriate medication use?	2011	Review

Supplementary Table 3: Quality assessment of included studies using Newcastle-Ottawa scale adapted for cohort and cross-sectional studies

Cohort study	Selection				Comparability[5]	Exposure			Total
	Representativeness[1]	Selection[2]	Ascertainment[3]	Demonstartion[4]		Assessment[6]	Duration[7]	Adequacy[8]	
Chandrasekhar D et al., 2019	1	1	1	1	1	1	1	1	8
Motallebzadeh N et al., 2019	1	1	1	1	1		1	1	7
Benjamin D et al., 2018	1	1	1	1	1	1	1	1	8
Devarapalli P et al., 2017	1	1	1	1	1	1	1	1	8
Kumar R et al., 2017	1	1	1	1	1	1	1	1	8
Narvekar R S et al., 2017	1	1	1	1	1	1	1	1	8
Anjum SM et al., 2017	1	1	1	1	1	1	1	1	8
Burla S et al., 2016	1	1	1	1	1	1	1	1	8
Kashyapa M et al., 2015	1	-	1	1	1	1	1	1	7
Pattani D et al., 2015	1	-	1	1	1	1	1	1	7
Umar NF et al., 2015	1	1	1	1	1	1	1	1	8
Undela K et al., 2013	1	1	1	1	1	1	1	1	8
Dhikav V et al., 2014	-	-	1	1	1	1	1	1	6
Momin TG et al., 2013	1	-	1	1	1	1	1	1	7
Vishwas HN et al., 2012	1	1	1	1	1	1	1	1	8
Shah RB et al., 2011	1	1	1	1	1	1	1	1	8
Mandavi et al., 2011	1	1	1	1	1	1	1	1	8
Zaveri HG et al., 2010	1	1	1	1	1	1	1	1	8
Harugeri A et al., 2010	1	1	1	1	1	1	1	1	8

Cross-sectional study	Selection				Comparability[5]	Outcome		Total
	Representativeness[9]	Selection [10]	Definition [11]	Assessment[12]		Assessment[13]	Statistical test[14]	
Bhatt AN et al., 2019	1	1	0	1	2	2	1	8
Pradhan S et al., 2018	1	1	1	1	2	2	1	9
Pradhan S et al., 2017	1	1	1	1	2	2	1	9

Borah L et al., 2017	-	-	1	1	-	2	-	4
Rakesh KB et al., 2017	1	1		1	2	2	-	7
Salwe K J et al., 2016	1	1	1	1	-	2	-	6
Chowta MN et al., 2016	-	-	-	1	2	2	-	5
Karandikar YS et al., 2013	1	-	-	2	2	2	1	8

1: Truly or somewhat representative of the exposed cohort; 2: Selection of the non-exposed cohort; 3: Assessment by structured interviews or surgical medical records; 4: Demonstration that outcome of interest was not present at start of study; 5: Study controls for the most important factor or any additional factor; 6: Assessment of outcome; 7: Follow-up long enough for outcomes to occur; 8: Adequacy of follow-up of cohorts. 9: sample representativeness; 10: sample selection procedure; 11: exposure definition; 12: risk factor exposure assessment; 13: Assessment of the outcome; 14: Statistical test is appropriate or not.

Supplemental Figure 1: Funnel plots

a. Polypharmacy use in older population

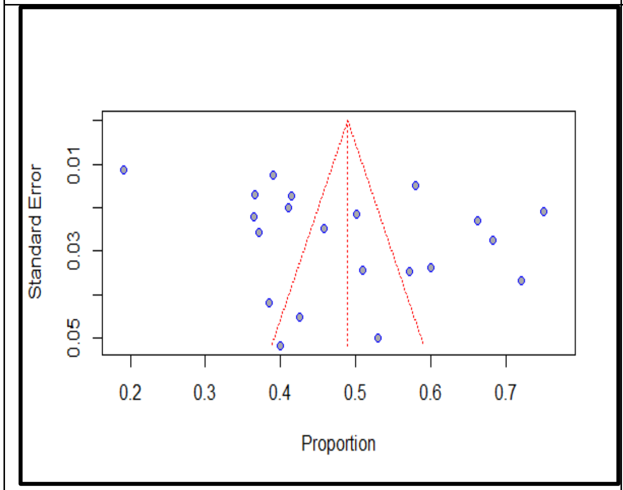

b. Hyperpolypharmacy in the older population

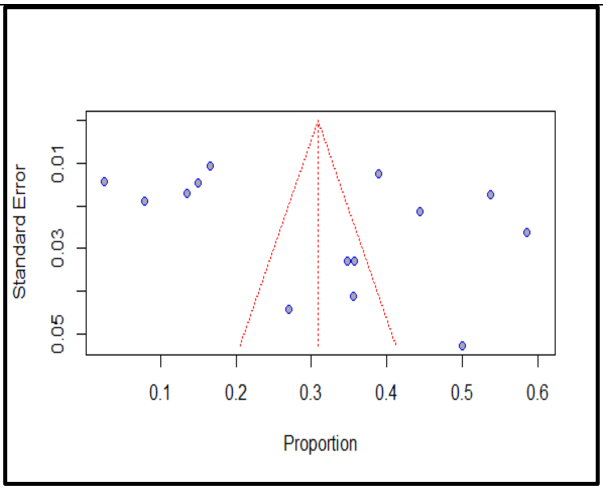

c. PIM use in older people

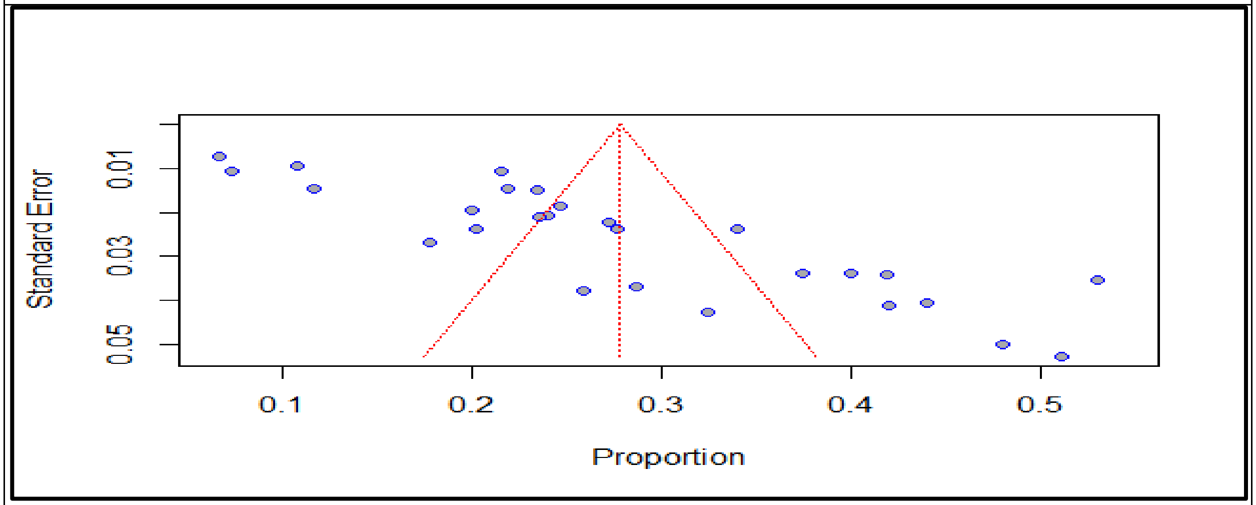

Egger and Begg tests

Outcome	Egger's test	Begg's test
Polypharmacy	0.034	0.304
Hyperpolypharmacy	0.211	0.352
Potential inappropriate medication use	0.027	0.001
